# The prognostic impact of t(11;14) in multiple myeloma: A real‐world analysis from the Australian Lymphoma Leukaemia Group (ALLG) and the Australian Myeloma and Related Diseases Registry (MRDR)

**DOI:** 10.1002/jha2.742

**Published:** 2023-07-25

**Authors:** Kenneth JC Lim, Cameron Wellard, Dipti Talaulikar, Joanne LC Tan, Joanna Loh, Pratheepan Puvanakumar, James A Kuzich, Michelle Ho, Matthew Murphy, Nicole Zeglinas, Michael SY Low, David Routledge, Andrew BM Lim, Simon D Gibbs, Hang Quach, Sue Morgan, Elizabeth Moore, Slavisa Ninkovic

**Affiliations:** ^1^ Department of Haematology St Vincent's Hospital Melbourne Melbourne Australia; ^2^ Victorian Cancer Cytogenetics Service St. Vincent's Hospital Melbourne Melbourne Australia; ^3^ School of Public Health and Preventive Medicine Monash University Melbourne Australia; ^4^ Department of Haematology The Canberra Hospital Canberra Australia; ^5^ Department of Medicine The Australian National University Canberra Australia; ^6^ Department of Haematology The Alfred Hospital Melbourne Australia; ^7^ Department of Haematology Monash Health Melbourne Australia; ^8^ Clinical Haematology Peter MacCallum Cancer Centre and Royal Melbourne Hospital Melbourne Australia; ^9^ Department of Haematology Austin Health and Olivia Newton John Cancer Research Institute Melbourne Australia; ^10^ Department of Haematology Eastern Health Melbourne Australia; ^11^ Department of Haematology Monash University Melbourne Australia; ^12^ Department of Medicine University of Melbourne Melbourne Australia

**Keywords:** BCL2, multiple myeloma, t(11;14), venetoclax

## Abstract

The prognostic impact of t(11;14) in multiple myeloma (MM) needs to be better understood to inform future treatment decisions. The Australian Lymphoma Leukaemia Group embarked on a retrospective, observational cohort study using real‐world data to interrogate treatment patterns and outcomes in 74 MM patients with t(11;14) [t(11;14)‐MM] diagnosed over 10 years. This was compared to 159 and 111 MM patients with high‐risk IgH translocations (IgH HR‐MM) and hyperdiploidy (Hyperdiploid‐MM), respectively, from the Australian Myeloma and Related Diseases Registry. No appreciable differences in age, gender, ISS, LDH levels, 1q21 or del(17p) status, or treatment patterns were observed between groups. Median PFS‐1 was not different between groups but both t(11;14)‐MM and IgH HR‐MM had an inferior PFS‐2 vs. Hyperdiploid‐MM: median PFS–2 8.2 months, 10.0 months, and 19.8 months (*p* = 0.002), respectively. The 3‐year OS were 69%, 71%, and 82% (*p* = 0.026), respectively. In the t(11;14)‐MM group, gain or amplification of 1q21 at diagnosis predicted for poorer OS (HR 3.46, *p* = 0.002). Eleven patients had received venetoclax with 45% achieving better than a very good partial response. Results suggest that t(11;14) MM may confer an unfavorable risk profile and that the use of targeted therapies such as venetoclax earlier in the treatment algorithm should be explored.

## 1 INTRODUCTION

Multiple myeloma (MM) is an incurable clonal plasma cell disorder characterized by the secretion of a monoclonal protein and/or serum or urinary free light chains and associated end organ dysfunction. The genetic profile and disease biology are heterogeneous leading to varied clinical outcomes. The introduction of multiple novel therapies including proteasome inhibitors (PIs), immunomodulatory agents (IMiDs), monoclonal antibodies (mAbs), antibody‐drug conjugates (e.g., belantamab mafodotin), selective nuclear export inhibitor (Selinexor), and, most recently, T‐cell‐directed immunotherapies such as bispecific T‐cell engagers and CAR‐T cells has not only prolonged survival for most patients but also expanded treatment options calling for clinicians to apply a risk‐adapted and targeted therapeutic approach. The revised International Staging System (R‐ISS) was developed by the International Myeloma Working Group (IMWG) to risk stratify newly diagnosed MM using a combination of biochemical markers and cytogenetic abnormalities [1]. Karyotyping and targeted fluorescence in situ hybridization (FISH) testing are integral to accurate identification of the key primary and secondary myeloma aberrations [2]. Primary events, seen as early as at the monoclonal gammopathy of uncertain significance (MGUS) stage come in two forms. The first involves a hyperdiploid karyotype characterized by gain of odd numbered chromosomes and is considered a standard‐risk primary genetic aberration. The other primary genetic aberration consists of a rearrangement of the immunoglobulin heavy chain (*IGH*) gene on the long arm of chromosome 14 with various partner oncogenes which are mutually exclusive of each other [2, 3]. The translocations involving *FGFR3*, *MAFA*, and *MAFB* genes on chromosomes 4, 16, and 20, respectively, confer a high‐risk profile, while rearrangement with *CCND1* on chromosome 11, that is, t(11;14), seen in about 20% of myeloma patients, is currently considered a standard‐risk primary genetic mutation [4, 5]. Gain in 1q and del(17p) are considered secondary high‐risk mutations (sHRM) and acquired after the primary event [4].

More recent studies have suggested, however, that t(11;14) MM may in fact respond poorly to currently used regimens which translate to inferior survival outcomes [6, 7, 8]. In addition, the t(11;14) abnormality also confers unique biology to malignant plasma cells in MM, demonstrating higher expression of antiapoptotic protein BCL‐2 as compared to other antiapoptotic proteins MCL‐1 and BCL‐xL [9]. In the intrinsic apoptotic pathway, anti–apoptotic proteins (i.e., BCL‐2, BCL‐xL, MCL‐1) are responsible for sequestering pro‐apoptotic ones (i.e., BAX, BAK, BIM, BID, NOXA) preventing cell death [9, 10]. As such, the associated higher proportional expression of BCL‐2 seen has allowed t(11;14) MM to emerge as a clinical relevant translocation which predicts response to BCL2‐inhibition. This is currently being investigated in a number of clinical studies of BCL2 inhibitors (BCL2i) including venetoclax, a potent, selective BCL‐2i that is approved for use in other hematological neoplasms including acute myeloid leukemia and chronic lymphocytic leukemia [11, 12, 13].

Given the increasing clinical relevance of t(11;14) in the era of BCL2i, and the conflicting reports of the prognostic impact of t(11;14), we sought to explore the outcome of patients harboring this translocation. The outcome of Australian MM patients with t(11;14) has not been reported on specifically and here we aim to report on the real‐world treatment and outcomes in this cohort of patients.

## 2 METHODS

### 2.1 Patients

A retrospective study was conducted by six participating sites from the Australasian Leukaemia and Lymphoma Group (ALLG). MM patients diagnosed between January 1, 2009, and December 31, 2019, harboring t(11;14) by either conventional karyotype or FISH analysis, were identified following interrogation of the Cytogenetics and FISH database of the Victorian Cytogenetic Service and The Canberra Hospital. Conventional karyotype was performed on non–enriched, non‐CD138 selected bone marrow aspirate samples. FISH analysis using the *CCND1::IGH* dual fusion probe was performed on PCs identified by cytoplasmic immunoglobulin staining with a positive cut‐off of 15%. Patient demographics, diagnostic, prognostic, treatment, and clinical outcome‐related data were extracted following review of electronic medical records. De‐identified patient‐level information collected comprised of structured data (patient demographics, laboratory test results, and treatment received) and unstructured data (physician notes, pathology reports, and minutes from multidisciplinary meetings) curated by the investigators.

The data set was subsequently compared with MM patients identified from the Myeloma and Related Diseases Registry (MRDR), which is a registry that contains prospectively collected data on patients with plasma cell dyscrasia diagnosed in Australia and NZ, now consisting over 6000 patients [14, 15]. Two groups of MM patients with varied primary genetic abnormalities were selected for comparison. A group consisting of patients with Hyperdiploid‐MM (Hyperdiploid‐MM) without t(11;14), t(4;14) or t(14;16) and a high‐risk IgH translocation (IgH HR‐MM) cohort consisting of patients with known t(4;14) or t(14;16). Patients diagnosed with MM from January 1, 2012, to December 31, 2019, were included. The status of secondary high‐risk mutations such as gain in 1q21 and deletion of 17p were accounted for in all three cohorts to ensure that there were no differences between groups. For completeness and transparency, patients with t(11;14) identified in the MRDR was compared to the ALLG t(11;14) cohort.

### 2.2 Outcome assessment

Treatment response assessments were made in accordance to the International Myeloma Working Group consensus criteria [16]. Best response outcomes were categorized as stringent complete response (sCR), complete response (CR), very good partial response (VGPR), partial response (PR), stable disease (SD), or progressive disease (PD). Overall survival (OS) was defined from the time of diagnosis to death from any cause. Progression‐free survival (PFS) was defined from the time of first treatment to time to disease progression or death from any cause. PFS of first‐line (PFS‐1), second‐line (PFS‐2), and third‐line (PFS‐3) regimens were obtained. Outcomes from venetoclax therapy were evaluated using event free survival (EFS), which was defined from the time of first treatment to time to disease progression, treatment cessation due to toxicity or death.

### 2.3 Statistical analysis

Statistical analyses were performed using GraphPad Prism v9.0 (GraphPad Software Inc., La Jolla, California, USA) and Stata 16.1. Univariate analyses of baseline demographics, clinical, and pathological variables were performed using Pearson's χ^2^ test and the Mann–Whitney *U* test. Kaplan–Meier curves were used for survival analysis (OS and PFS) and when required, landmark analysis was performed to reduce immortality bias. Comparisons between groups were analyzed using the log‐rank (Mantel‐Cox) test and univariate Cox proportional hazards regression models. A *p* value of < 0.05 was considered statically significant.

## 3 RESULTS

### 3.1 Patient characteristics

#### 3.1.1 t(11;14)‐MM cohort from the ALLG

Patient characteristics are summarized in Table 1. Seventy‐four patients with t(11;14) MM were identified: 40 (54%) by conventional karyotype only, 24 (32%) by FISH only, and 10 (14%) by both conventional karyotype and FISH. Thirty‐eight (51%) were diagnosed with MM before 2015. The median age at time of diagnosis was 65 years (IQR 56.0, 72.0). By ISS, 43% (29/67) were classified as high risk (Stage III). R‐ISS was available for 52 patients with five (9.6%) defined as high risk. Additional cytogenetic aberrations were identified in 61 (82%) patients. Of relevance, monosomy 13/deletion of 13q was seen in 30 patients (41%), gain (3 copies) and/or amplification (> 3 copies) of 1q in 9 (12%), and deletion of 17p in 8 (11%). Forty‐nine (92%) patients had an ECOG of < 2 and 47 (64%) of patients were classified as transplant eligible.

**TABLE 1:** Baseline characteristics of patients in the different primary cytogenetics risk groups.

Factor	IgH HR‐MM^a^	Hyperdiploid‐MM^a^	T(11;14)‐MM^b^	*p* Value
*N*	159	111	74	
Age Dx, median (IQR)	61.8 (53.6, 70.0)	64.6 (59.7, 72.5)	65.0 (56.0, 72.0)	
Age ≥ 65	71/159 (44.7%)	54/111 (48.6%)	40/74 (54%)	0.49
Gender				0.36
Male	88/159 (55.3%)	71/111 (64.0%)	43/74 (58.1%)	
Female	71/159 (44.7%)	40/111 (36.0%)	31/74 (41.9%)	
ISS = 3	50/127 (39.4%)	28/93 (30.1%)	29/67 (43.2%)	0.44
R‐ISS = 3	44/123 (35.8%)	7/81 (8.6%)	5/52 (9.6%)	<0.001
ECOG ≥ 2	14/100 (14.0%)	17/61 (27.9%)	4/50 (8.0%)	0.013
LDH > ULN	29/124 (23.4%)	22/89 (24.7%)	19/63 (30.2%)	0.59
ASCT	104/145 (71.7%)	65/103 (63.1%)	43/74 (58.1%)	0.10
1q21	22/159 (13.8%)	22/111 (19.8%)	9/66 (13.6%)	0.36
Del(17p)	14/159 (8.8%)	8/111 (7.2%)	8/66 (12.1%)	0.54
Del(13q)	N/A	N/A	30/66 (45%)	

Abbreviations: ASCT, autologous stem cell transplant; ECOG, European Cooperative Oncology Group; ISS, International Staging System; LDH, lactate dehydrogenase; R‐ISS, revised International Staging System.

^a^ Data from the Myeloma and Related Diseases Registry.

^b^ Data from the Australian Lymphoma and Leukaemia Group.

#### 3.1.2 Hyperdiploid‐MM and IgH HR‐MM cohort from the MRDR

There were 270 MRDR cases diagnosed during a similar time frame identified: 159 patients with IgH HR‐MM [t(4;14) or t(14;16)] and 111 patients with Hyperdiploid‐MM. Baseline characteristics are presented together with the ALLG t(11;14) cohort in Table 1. There were no appreciable differences in age, gender, ISS, LDH levels, 1q21, or del(17p) status between the two groups and the ALLG t(11;14) cohort. The Hyperdiploid‐MM cohort had a significantly higher proportion of patients with ECOG ≥ 2 (*p* = 0.013). No appreciable differences between first‐line and second‐line treatment regimens were found amongst the groups; treatment details are summarized in Tables S1 and S2.

### 3.2 Treatment outcomes: ALLG t(11;14) multiple myeloma cohort

Patient outcomes by line of therapy are summarized in Table 2. The median follow‐up from time of MM diagnosis was 5.22 years (range 0.14–10.32). The median OS was 5.4 years (95% CI 4.1–6.6) and the 3‐year OS was 69% (95% CI 57–79).

**TABLE 2:** Outcomes in patients by line of therapy in t(11;14) cohort.

	First line	Second line	Third line
Total number of patients	*n* = 74	*n* = 47	*n* = 32
PI based	60 (81%)	11 (23%)	8 (25%)
IMiD based	10 (14%)	18 (38%)	12 (38%)
PI and IMiD combination	0 (0%)	4 (9%)	2 (6%)
Monoclonal Ab combination	3 (4%)	5 (11%)	4 (13%)
Chemotherapy	0 (0%)	5 (11%)	4 (13%)
Other novel agents	1 (1%)	4 (9%)	2 (6%)
Consolidative autograft	43 (58%)		
Maintenance therapy	30 of 43 (70%)		
ORR	86%	49%	56%
VGPR or better	38%	28%	22%
3‐year PFS (95% CI)	0.30 (0.19–0.42)		
Median PFS, months (95% CI)	22.1 (18.7–29.4)	8.2 (4.0–14.3)	5.9 (3.7–13.0)

Abbreviations: Ab, antibody; IMiD, immunomodulatory drugs; MRR, major response rate; ORR, overall response rate; PFS, progression‐free survival; PI, proteasome inhibitor.

*Note*: Data from the Australian Lymphoma and Leukaemia Group.

#### 3.2.1 Outcomes of first‐line therapy

Sixty‐three (85%) patients received PI‐based treatment with the majority (56/63) receiving VCd (bortezomib, cyclophosphamide, and dexamethasone), four receiving Vd, two receiving VCd with daratumumab, and one receiving VCd with venetoclax. Ten (14%) patients received an IMiD‐based first‐line therapy: six lenalidomide/dexamethasone (Rd) and four cyclophosphamide, thalidomide, and dexamethasone (CTd). Forty‐seven patients (63%) were deemed transplant‐eligible: 43 (58%) proceeded with an upfront autologous stem cell transplant (ASCT), 1 had rapidly progressive disease, and 3 had declined proceeding with ASCT. IMiD‐based maintenance therapy was delivered in 70% (30/43) of patients.

The overall response rate (ORR) was 86% with 38% achieving a VGPR or better. With a median follow‐up of 5.22 (range 0.14–10.32) years, median PFS‐1 was 1.91 years (95% CI 1.7–2.6). The difference between PFS‐1 for PI‐based induction (*n* = 63) and IMiD‐based regimens (*n* = 10) was not significant [median 1.84 vs. 4.58 years; HR 1.47 (0.61–3.52)]. ORR after first‐line therapy (ASCT inclusive) was higher in patients who received ASCT (*n* = 43) than those who did not (*n* = 31) [95% (41/43) vs. 74% (23/31), *p* = 0.0139]. Median time to ASCT was 7.33 (IQR 5.39–8.66) months. Through landmark analysis (landmark time was 7.33 months after start of first‐line treatment), PFS‐1 and OS were significantly longer in patients who underwent ASCT compared to patients who did not receive upfront ASCT; median PFS‐1 was 2.02 vs. 0.87 years (HR 0.41; CI 0.20–0.85; *p* = 0.001) and median OS was 5.87 vs. 1.42 years (HR 0.29; 95% CI 0.13–0.68; *p* < 0.001).

#### 3.2.2 Outcomes in relapsed/refractory MM

Second‐ and third‐line therapy was commonly IMiD‐based (40% and 38%, respectively) with incorporation of anti‐CD38 monoclonal antibodies (mAbs) (second‐line 10%, third‐line 13%). Median PFS‐2 was 0.77 years (95% CI 0.39–0.98) while median PFS‐3 was 0.65 years (95% CI 0.34–1.16).

#### 3.2.3 1q gain/amplification confers higher risk in t(11;14) myeloma

In an exploratory univariate analysis, no significant differences in OS were observed with ISS III vs. ISS I/II (HR 1.27; 95% CI 0.63–2.53; *p* = 0.492), age ≥65 vs. < 65 years (HR 1.81; 95% CI 0.92–3.55), and elevated LDH vs. normal LDH (HR 1.52; 95% CI 0.69–3.37; *p* = 0.245; see Table 3). Patients with 1q gain and/or 17p had significantly poorer OS than those with no secondary HRM (HR 2.34; 95% CI 0.95–5.71; *p* = 0.015). In a subset analysis, patients with a gain or amplification of 1q (*n* = 9) had a poorer OS compared to patients without sHRM (*n* = 54) with a median OS of 2.0 vs. 6.2 years, respectively (HR 3.46; 95% CI 0.93–12.02; *p* = 0.002). In contrast, OS was not significantly different between patients with del(17p) vs. those without sHRM (*n* = 29) (HR 1.78; 95% CI 0.59–5.11; *p* = 0.22).

**TABLE 3:** Effect of baseline characteristics on OS in the t(11;14) group.

	**Overall survival (OS)**	
	HR	*p* Value
Age ≥65 vs. < 65 years	1.81 (0.92–3.55)	0.56
LDH elevated	1.52 (0.69–3.37)	0.245
ISS III vs. ISS I/II	1.27 (0.63–2.53)	0.492
1q gain and/or 17p vs. no secondary HRM	2.34 (0.95–5.71)	0.015

Abbreviations: HRM, high‐risk mutation; ISS, International Staging System; LDH, lactate dehydrogenase.

*Note*: Data from the Australian Lymphoma and Leukaemia Group.

#### 3.2.4 Response to venetoclax

Eleven patients [median 3 prior lines of therapy (range 0–9)] were exposed to venetoclax. Of these patients, six (55%) received venetoclax in combination with a PI, three (27%) as a triplet (PI and mAb), and two (18%) as monotherapy in combination with dexamethasone. ORR to venetoclax‐based therapy was 55% with 45% achieving a VGPR or better. Median EFS with venetoclax was 0.54 years (95% CI 0.05–2.17). Median EFS for patients with 1–4 lines (*n* = 6) was 1.22 years and median EFS for patients with 5 lines or more (*n* = 5) was 0.54 years (HR 0.56; 95% CI 0.12–2.5; *p* = 0.54).

### 3.3 Treatment outcomes: comparison with the MRDR cohorts

Thirty nonoverlapping t(11;14) patients were identified in the MRDR cohort. There were no significant differences between OS, PFS‐1, and PFS‐2 detected between the ALLG and MRDR t(11;14) cohorts (survival curves presented in Figures S1–S3).

**FIGURE 1:**
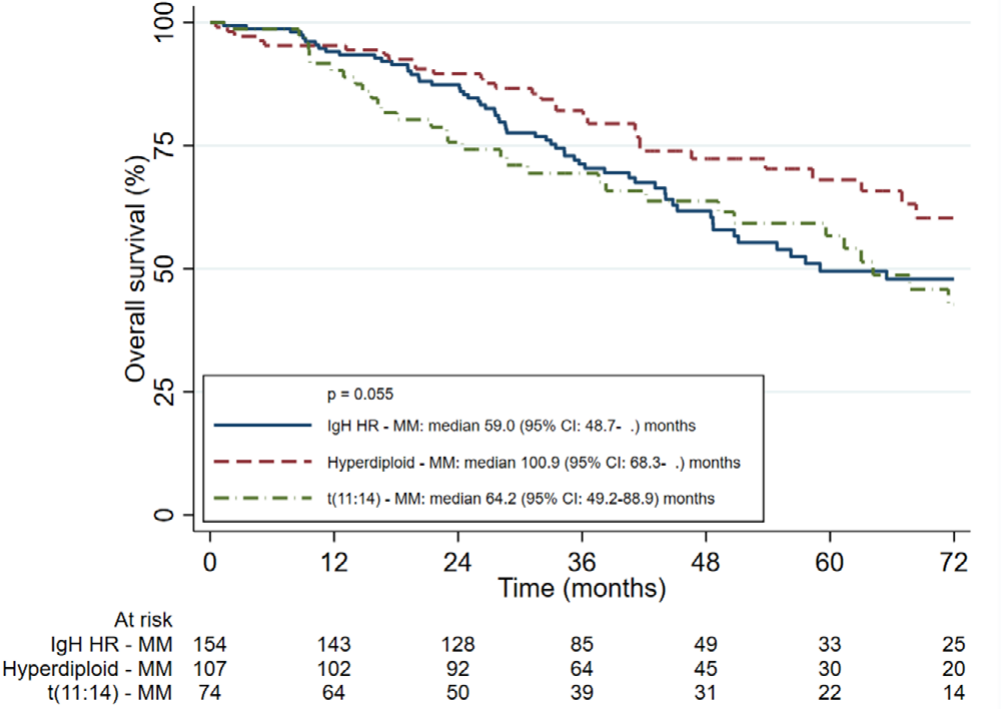
Overall survival curves of IgH high‐risk, hyperdiploid, and t(11;14) groups.

**FIGURE 2:**
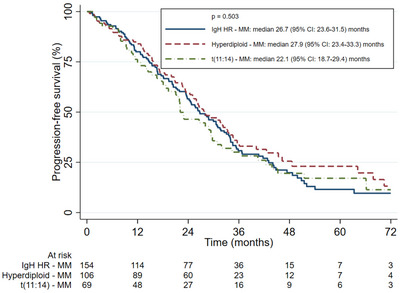
Progression‐free survival curves after first‐line therapy of the IgH high‐risk, hyperdiploid, and t(11;14) groups.

**FIGURE 3:**
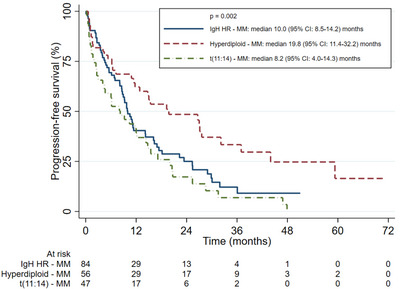
Progression‐free survival curves after second‐line therapy of the IgH high‐risk, hyperdiploid, and t(11;14) groups.

The ALLG cohort was compared to the IgH HR‐MM and Hyperdiploid‐MM cohorts from the MRDR. There were no differences in ORR for first‐line [IgH HR‐MM 84%, Hyperdiploid‐MM 83%, t(11;14)‐MM 86%; (*p* = 0.81)] nor second‐line [IgH HR‐MM 32%, Hyperdiploid‐MM 40%, t(11;14) 49%; (*p* = 0.22)] therapy. There were no significant differences in OS between the ALLG t(11;14)‐MM and IgH HR‐MM cohorts [3‐year OS 69% (95% CI 57–79) vs. 71% (95% CI 63–78)] but both had a poorer OS to the Hyperdiploid‐MM cohort [3‐year OS 82% (95% CI 73–88); *p* = 0.026]; OS curves are shown in Figure 1. There was no significant difference in PFS‐1 between groups [median PFS‐1: IgH HR‐MM 26.7 months (95% CI 23.6–31.5), Hyperdiploid‐MM 27.9 months (95% CI: 23.4–33.3), t(11;14)‐MM 22.1 months (95% CI 18.7–29.4); *p* = 0.503]. Both the t(11;14)‐MM and IgH HR‐MM cohort had an inferior PFS‐2 compared to the Hyperdiploid‐MM cohort [median PFS‐2: t(11;14)‐MM 8.2 months (95% CI 4.0–14.3), IgH HR‐MM 10.0 months (95% CI 8.5–14.2), Hyperdiploid‐MM 19.8 months (95% CI 11.4–32.2); *p* = 0.002]. PFS‐1 and PFS‐2 curves are shown in Figures 2 and 3.

## 4 DISCUSSION

This study is the first to characterize Australian practice in the diagnosis and management of t(11;14) MM in the last decade (2009–2019). Overall, characteristics of patients with t(11;14), including ISS stage and cooccurrence of secondary high‐risk mutations (1q gain/amplification and 17p deletion) are comparable to local registry data and international retrospective studies [7, 14].

Currently, both the revised International Staging System and the updated mSMART consensus guidelines still consider patients with isolated t(11;14) as standard risk [1, 17]. However, our study demonstrates that in the era of IMiDs, PIs, and anti‐CD38 mAb, survival patterns of t(11;14) MM appear inferior to standard‐risk MM and may mirror that of the high‐risk cohort. More than 10 retrospective studies have so far been performed attempting to answer this question with variable results [18]. The largest so far of 3498 patients showed that t(11;14) with no other HR abnormality (*n* = 589) had a negative impact on PFS‐1 when compared to non‐t(11;14) with no HR abnormality (t(4;14), t(14;16), t(14;20), del17p, or gain in 1q) [19]. In the same study, concurrent t(11;14) with del(17p) also resulted in worse OS compared to del (17p) without IgH translocations suggesting a possible “double‐hit” effect. Conversely, a subsequent large prospective observational cohort study including 378 t(11;14) MM patients found no PFS or OS disadvantage in t(11;14) MM against a standard‐risk cohort [20].

No PFS differences between our cohorts after front‐line therapy were observed suggesting that the prognostic impact of t(11;14) may not be as pronounced in a single novel agent front‐line strategy given the lack of PI+IMiD combinations in our t(11;14) MM cohort. Kaufman et al.’s retrospective study on patients receiving VRd induction therapy showed that patients with t(11;14) had lower rates of ≥VGPR postinduction and shorter median PFS when compared to non‐t(11;14) patients [21]. In contrast, another retrospective study including a mixture of patients receiving single and dual novel agent induction (including 40%–50% receiving VRd) showed poorer TTNT (time to next treatment) in their high‐risk group but no difference between the t(11;14) and standard‐risk groups. However, subgroup analysis of the VRd‐treated subgroup showed shorter TTNT for the t(11;14) group compared to the standard‐risk group suggesting the blunted impact of PI + IMiD in treating t(11;14) MM in the front‐line setting [22]. Consistent with other studies, upfront ASCT in transplant‐eligible patients likely remains important in t(11;14) MM with our analysis showing higher ORR and longer PFS‐1 in patients who underwent ASCT vs. those that did not [7, 23, 24]. Gao et al. in their retrospective of 455 patients further confirmed that differences in ≥VGPR rates before ASCT evened out after ASCT between t(11;14), standard‐risk, and high‐risk groups translating to the lack of PFS difference between the t(11;14) and standard‐risk group.

In our study, there was an appreciable survival difference after second‐line therapy with the t(11;14)–MM and IgH HR–MM cohorts showing poorer PFS vs. the Hyperdiploid–MM group suggesting that t(11;14) may in fact be a poor prognostic marker in the relapsed/refractory setting. One reason is the greater incidence of acquired secondary high‐risk mutations (such as del(17p) and chromosome 1 abnormalities) due to increased genomic instability [25]. Co–occurrence of these secondary mutations with t(11;14) has also been shown to negatively impact survival outcomes similar to that of t(4;14) MM [19]. Currently, del(17p) has been consistently shown to impact survival in t(11;14) MM with conflicting evidence in chromosome 1 abnormalities [7, 19, 24, 26]. In our univariate analysis, gain in 1q was found to have a negative impact on OS. With the increasing use of monoclonal antibodies, second‐generation IMiDs, and PIs in the relapsed/refractory setting, it is hypothesized that these therapies may be insufficient to abrogate the negative impact of t(11;14). This emphasizes the need to develop targeted therapies for t(11;14) MM in the relapsed refractory setting. Currently, a Phase III RCT is underway examining venetoclax against pomalidomide in combination with dexamethasone in R/R MM [27]. Notably, the Phase III BELLINI study of venetoclax, bortezomib, dexamethasone (VenVd), versus Vd found ≥VGPR rates of 70% in t(11;14) patients receiving VenVd and an OS advantage against Vd in their subgroup analysis [13]. Another venetoclax‐based combination being explored is Ven‐Dara‐Dex (VenDd), showing promising early outcomes with ≥VGPR rates of >90% in R/R MM [28, 29]. In our exploratory analysis of venetoclax use in our t(11;14) cohort, deep responses were achieved in nearly half the cohort but responses were not sustained in later lines of therapy. While this study was not powered to determine the optimal timing of venetoclax, there is suggestion that earlier use of venetoclax in t(11;14) MM may be beneficial for optimizing efficacy [11, 30, 31]. As such, future directions point towards the continued investigation of early‐line combination strategies incorporating a BCL‐2 inhibitor (i.e., with anti‐CD38 mAb or PI) particularly in the front‐line or maintenance therapy setting. In addition, clinical trials involving second‐generation BCL‐2 inhibitors are underway and we await their results with interest [32]. The role of more novel therapeutic agents, such as T‐cell‐directed therapy, still needs to be further examined in this patient group.

Our study has several limitations expected in retrospective studies including the burden of dealing with missing data. FISH for t(11;14) was not consistently performed in our cohort as it was not routine practice in Australia during the time period of the study. A sizeable proportion of our cases were identified by conventional karyotyping of unsorted bone marrow samples. The poorer sensitivity of this method may have allowed cases with lower disease burden or cryptic translocations to be missed causing a selection bias toward a more proliferative, high‐risk cohort of t(11;14) cases. Comparison of our cohort with registry data was limited due to differences in patient selection processes; however, this was overcome by ensuring that there were no significant differences between groups, including nonoverlapping patients from the MRDR t(11;14) cohort, in confounding factors such as age, ISS, performance status, secondary high‐risk mutations, and treatment received.

The long duration of our study transcended FDA approval of many antimyeloma agents resulting in a treatment paradigm shift [7, 8, 16–19]. This likely explains the pattern of first‐line therapy in our study, which ranged from doublet regimens such Rd and VD to quadruplet regimens adding newer agents such as daratumumab and venetoclax to a VCD backbone. No IMiD and PI containing triplet first‐line regimens were used given that VRd induction only became available in Australia since June 2020. This is substantially different from other international cohorts, which contain a high proportion of patients treated with IMiD and PI combinations during this period [18, 20, 21]. The impact of these differences in treatment landscape on outcomes of our t(11;14) cohort of patients is unknown and hence direct comparisons to other international t(11;14) cohorts during this time period should be interpreted with caution.

## 5 CONCLUSION

In conclusion, our study provides insight into the management of t(11;14) MM in Australia in the era of IMiDs, PIs, and anti‐CD38 mAbs. In our t(11;14)‐MM cohort, OS is inferior to that of Hyperdiploid‐MM and may in fact follow the trajectory of MM with high‐risk IgH translocations due to shorter sustained responses to available therapies in the relapsed/refractory setting. Secondary mutations such as gain or amplification in 1q21 appear to negatively impact survival in this group of patients and confer an adverse‐risk profile. Venetoclax is a promising targeted therapy, particularly when used earlier in the treatment algorithm. Correct and early identification of this mutation is, hence, important as we move away from a one‐size‐fits‐all approach toward more targeted and risk‐directed strategies.

## AUTHOR CONTRIBUTIONS

Kenneth J.C. Lim and Slavisa Ninkovic designed the study. Kenneth JC Lim, Joanne LC Tan, Joanna Loh, Pratheepan Puvanakumar, James A Kuzich, Michelle Ho, Matthew Murphy, Nicole Zeglinas, and Elizabeth Moore collected and assembled the data. Kenneth J.C. Lim and Cameron Wellard analysed the data. Slavisa Ninkovic provided study supervision. Kenneth J.C. Lim and Slavisa Ninkovic drafted the manuscript. All authors: revised and approved the final manuscript.

## CONFLICT OF INTEREST STATEMENT

The MRDR has received funding from Abbvie, Amgen, Antengene, Bristol‐Myers Squibb, Celgene, Gilead, GSK, Janssen, Novartis, Sanofi, and Takeda.

## FUNDING INFORMATION

This study did not receive any external funding.

## ETHICS STATEMENT

This study has received approval from the St. Vincent's Hospital Human Research Ethics Committee (HREC; low‐risk pathway), Melbourne, Australia.

## PATIENT CONSENT STATEMENT

HREC approved a waiver of consent for data linkage of patient records using personal identifiers. Once the data were linked, it was analyzed with personal identifiers removed.

## CLINICAL TRIAL REGISTRATION

The authors have confirmed clinical trial registration is not needed for this submission.

## Supporting information

Supporting Information

## Data Availability

The data that support the findings of this study are available from the corresponding author upon reasonable request. For original data, please contact kenneth.lim@svha.org.au
