# Supplementary material for: The prognostic impact of t(11;14) in multiple myeloma: A real‐world analysis from the Australian Lymphoma Leukaemia Group (ALLG) and the Australian Myeloma and Related Diseases Registry (MRDR)
Source: EJHaem. 2023 Jul 25;4(3):639–46. doi: 10.1002/jha2.742 (PMC10435683; doi:10.1002/jha2.742)
Supplement: Supplementary file 1 — Supporting Information [file JHA2-4-639-s001.docx]

**Supplement Table 1: Details of first-line therapy by cytogenetic risk groups**

| Factor | IgH HR-MM^a^ | Hyperdiploid-MM^a^ | T(11;14)-MM^b^ | p-value |
| --- | --- | --- | --- | --- |
| Regimen |  |  |  | 0.17 |
| PI | 131/152 (86.2%) | 89/107 (83.2%) | 60/74 (81.1%) |  |
| IMiD | 8/152 (5.3%) | 10/107 (9.3%) | 10/74 (13.5%) |  |
| PI + IMiD | 9/152 (5.9%) | 3/107 (2.8%) | 0/74 (0.0%) |  |
| aCD38 + PI | 3/152 (2.0%) | 5/107 (4.7%) | 3/74 (4.1%) |  |
| ORR | 118/141 (83.7%) | 78/94 (83.0%) | 64/74 (86.4%) | 0.81 |

Abbreviation: PI, proteasome inhibitor; IMiD, immunomodulatory drugs; aCD38, anti-CD38 antibody; ORR; Overall response rate; IgH HR-MM, IgH translocation (high-risk) Multiple Myeloma; Hyperdiploid-MM, hyperdiploid Multiple Myeloma; T(11;14)-MM, t(11;14) Multiple Myeloma

^a^ data from the Myeloma and related diseases registry

^b^ data from the Australian Lymphoma and Leukaemia group

**Supplement Table 2: Details of second-line therapy by cytogenetic risk groups**

| Factor | IgH HR-MM^a^ | Hyperdiploid-MM^a^ | T(11;14)-MM^b^ | p-value |
| --- | --- | --- | --- | --- |
| Regimen |  |  |  | 0.13 |
| PI | 15/77 (19.5%) | 7/54 (13.0%) | 11/47 (23.4%) |  |
| IMiD | 43/77 (55.8%) | 38/54 (70.4%) | 18/47 (38.3%) |  |
| PI + IMiD | 3/77 (3.9%) | 1/54 (1.9%) | 4/47 (8.5%) |  |
| aCD38 +/- other drug | 16/77 (20.8%) | 8/54 (14.8%) | 5/47 (10.6%) |  |
| ORR | 19/59 (32.2%) | 16/40 (40.0%) | 23/47 (49%) | 0.22 |

Abbreviation: PI, proteasome inhibitor; IMiD, immunomodulatory drugs; aCD38, anti-CD38 antibody; ORR; Overall response rate; IgH HR-MM, IgH translocation (high-risk) Multiple Myeloma; Hyperdiploid-MM, hyperdiploid Multiple Myeloma; T(11;14)-MM, t(11;14) Multiple Myeloma

^a^ data from the Myeloma and related diseases registry

^b^ data from the Australian Lymphoma and Leukaemia group

**Supplement Figure 1: Overall Survival curves of the ALLG t(11;14)-MM and MRDR t(11;14)-MM Cohorts**

**
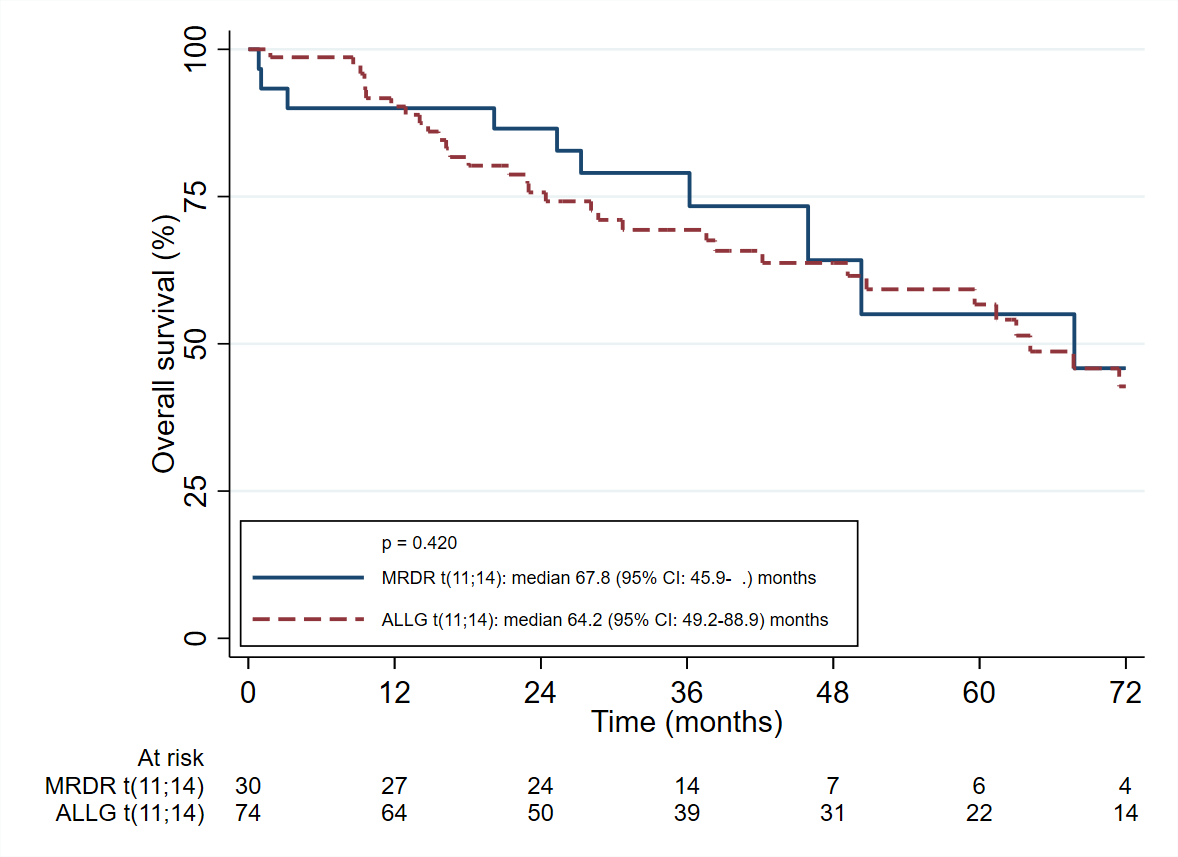
**

**Supplement Figure 2: Progression free survival curves after 1^st^ line therapy of the ALLG t(11;14)-MM and MRDR t(11;14)-MM Cohorts**

**
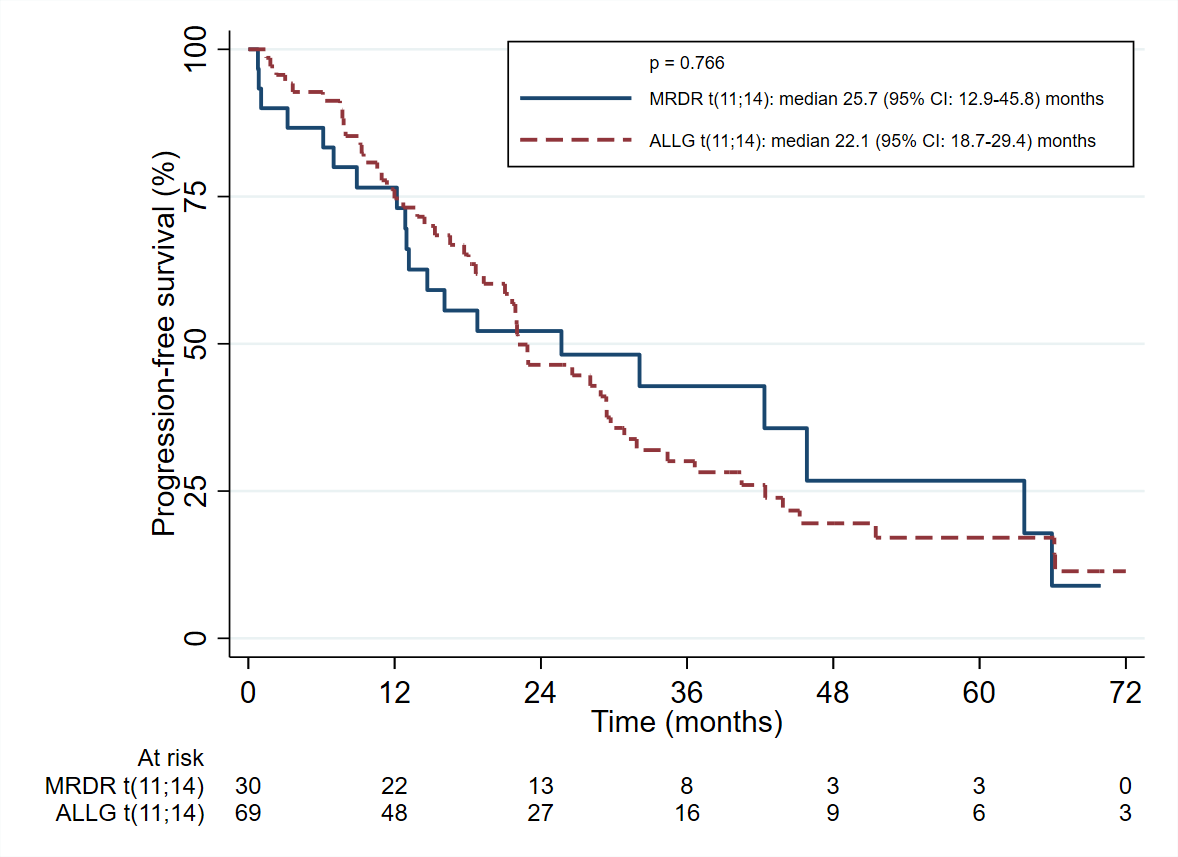
**

**Supplement Figure 3: Progression free survival curves after 2^nd^ line therapy of the ALLG t(11;14)-MM and MRDR t(11;14)-MM Cohorts**

**
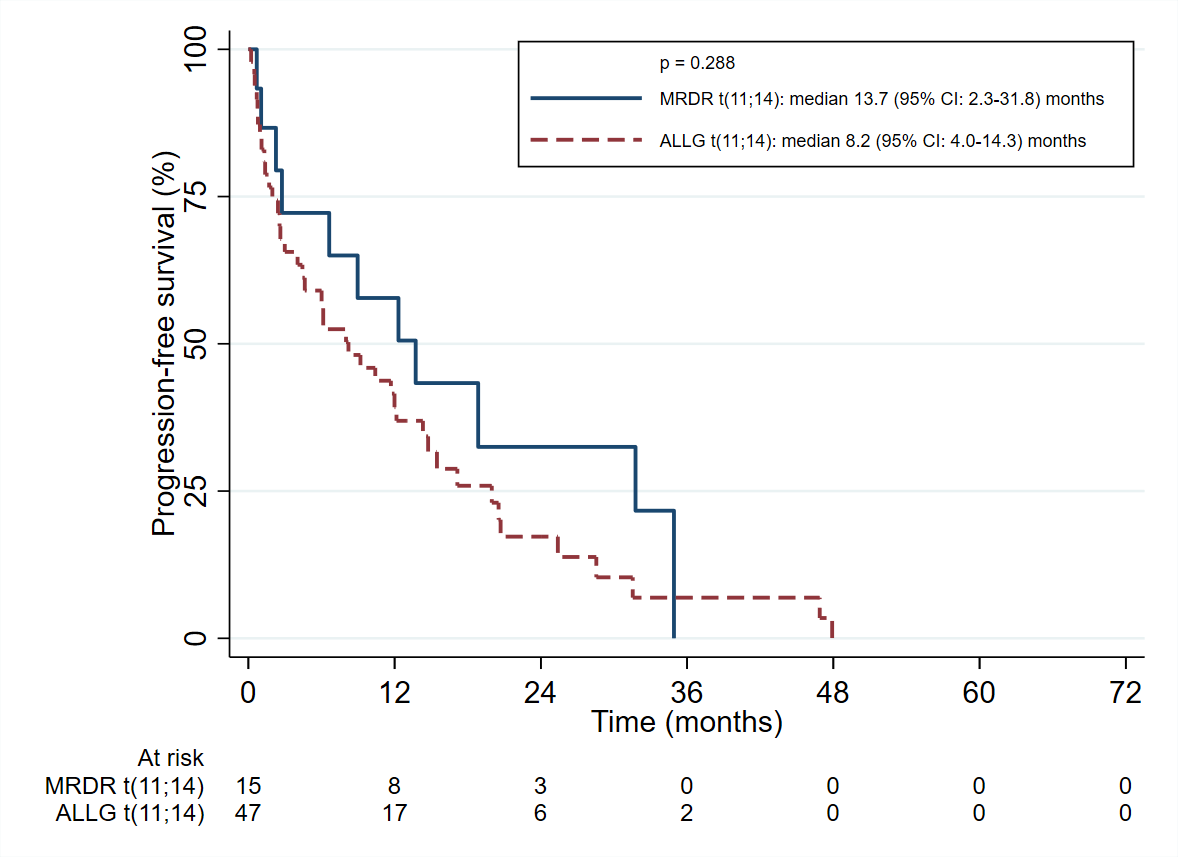
**
